# A Compact Closed-Loop Optogenetics System Based on Artifact-Free Transparent Graphene Electrodes

**DOI:** 10.3389/fnins.2018.00132

**Published:** 2018-03-06

**Authors:** Xin Liu, Yichen Lu, Ege Iseri, Yuhan Shi, Duygu Kuzum

**Affiliations:** Neuroelectronics Group, Department of Electrical and Computer Engineering, University of California, San Diego, La Jolla, CA, United States

**Keywords:** optogenetics, graphene, closed-loop optogenetics, transparent graphene array, multi-electrode array, light-induced artifact, neural recordings, electrophysiology

## Abstract

Electrophysiology is a decades-old technique widely used for monitoring activity of individual neurons and local field potentials. Optogenetics has revolutionized neuroscience studies by offering selective and fast control of targeted neurons and neuron populations. The combination of these two techniques is crucial for causal investigation of neural circuits and understanding their functional connectivity. However, electrical artifacts generated by light stimulation interfere with neural recordings and hinder the development of compact closed-loop systems for precise control of neural activity. Here, we demonstrate that transparent graphene micro-electrodes fabricated on a clear polyethylene terephthalate film eliminate the light-induced artifact problem and allow development of a compact battery-powered closed-loop optogenetics system. We extensively investigate light-induced artifacts for graphene electrodes in comparison to metal control electrodes. We then design optical stimulation module using micro-LED chips coupled to optical fibers to deliver light to intended depth for optogenetic stimulation. For artifact-free integration of graphene micro-electrode recordings with optogenetic stimulation, we design and develop a compact closed-loop system and validate it for different frequencies of interest for neural recordings. This compact closed-loop optogenetics system can be used for various applications involving optogenetic stimulation and electrophysiological recordings.

## Introduction

Electrophysiology has been the backbone of neuroscience research for decades. The last decade has witnessed rapid advancements in multi-photon imaging methods for monitoring hundreds of cells densely packed in neuronal microcircuits with high resolution. The advent of optogenetics has revolutionized neuroscience research by enabling selective control of neural activity and casual manipulation of specific neural circuits. Crosstalk-free integration of optical imaging, optogenetics and electrophysiological recordings can transform spatiotemporal mapping of neural circuits and can allow unprecedented studies of functional neural connectivity. However, conventional metal-based microelectrodes are not suitable for that purpose since they suffer from prominent light-induced artifacts generated by optical imaging or stimulation. Therefore, a new generation of optically transparent neural probes which eliminate light-induced artifact problem is needed. Several transparent microelectrode arrays based on ITO (Gross et al., 1985; Ledochowitsch et al., 2011; Kwon et al., 2013) and graphene (Kuzum et al., 2014; Park et al., 2014) have been demonstrated. Graphene electrodes hold a great promise for neural monitoring applications, owing to the unique combination of properties including transparency, flexibility (Lee et al., 2008), high conductivity (Geim and Novoselov, 2007), biocompatibility (Li et al., 2013; Sahni et al., 2013), and single-molecule level sensitivity (Schedin et al., 2007). Transparent microelectrode arrays made of graphene have been used for multimodal probing of neural circuits using two-photon microscopy (Kuzum et al., 2014) and optogenetics (Park et al., 2014). The optical transparency of graphene has been shown to enable efficient delivery of light for imaging of neuronal populations with high spatial resolution while recording their neural activity by the graphene electrode with high temporal resolution.

All closed-loop optogenetics systems demonstrated to-date use conventional metal-based electrodes (Krook-Magnuson et al., 2013; Paz et al., 2013; Siegle and Wilson, 2014; Pashaie et al., 2015). Light-induced artifacts in conventional metal electrodes appear as transients or oscillations in recordings and can interfere with local field potentials or spike recordings, depending on the frequency and duration of the light stimulus. It is not easy to distinguish these artifacts from real neural activity, especially in the local field potential (LFP) range, which includes important information on cortical dynamics. Since these artifacts can cause false-positives during real-time closed-loop operation, additional steps will be required to detect and remove those artifacts from the neural recordings, which will increase the complexity of the closed-loop control system. Therefore, elimination of the artifacts is critical to simplify the data processing for closed-loop operation and to achieve temporal precision in stimulation. Unlike metals, graphene has great potentials to solve the artifact problem. The photo-induced currents are intrinsically very weak and fast in graphene, requiring special structures or extremely low temperatures to even detect them (Gabor et al., 2011; Lemme et al., 2011). Therefore, use of graphene arrays in the closed-loop optogenetics system brings significant advancements through direct elimination of light-induced artifacts. Besides the light-induced artifact problem, current closed-loop optogenetics systems are generally tethered to bulky equipment since they use computers to process electrophysiological data online for controlling the light source. Physical tethers impede movement, limit animal behavior in complex environments and significantly degrade the longevity of chronic experiments. A portable and small-sized closed-loop optogenetics system is crucial for reliable long-term studies in awake animals. Long-term studies of closed-loop optogenetics in animal models can facilitate the investigation of local neural circuit connections (Stark et al., 2014). It can also help to identify the networks and cell types involved in various neurological disorders (Krook-Magnuson et al., 2013; Pashaie et al., 2015).

Here, we present a compact battery-powered closed-loop optogenetics system enabled by transparent graphene electrode array. The graphene array was fabricated on clear flexible polymer substrates, which are optically transparent at a broad range of wavelengths used for optogenetics. Graphene transfer and subsequent fabrication steps were optimized to achieve high yield in large area graphene multielectrode arrays. Light-induced artifacts were investigated for graphene electrodes and Au control electrodes for different light stimulation intensities and durations. An equivalent circuit model was established to explain the recording of light-induced artifacts in Au electrodes. An optical stimulation module including micro-LED chips coupled to optical fibers to deliver the light stimulation directly to target depth was developed. A hardware system was designed to combine the micro-electrode and optical module to form the closed-loop operation. This system was tested for artificial signals of various frequencies, durations and waveforms that resemble the biological ones. Finally, algorithms that can be implemented in this closed-loop system and possible hardware improvements for the system design are discussed.

## Materials and methods

**Figure 1:**
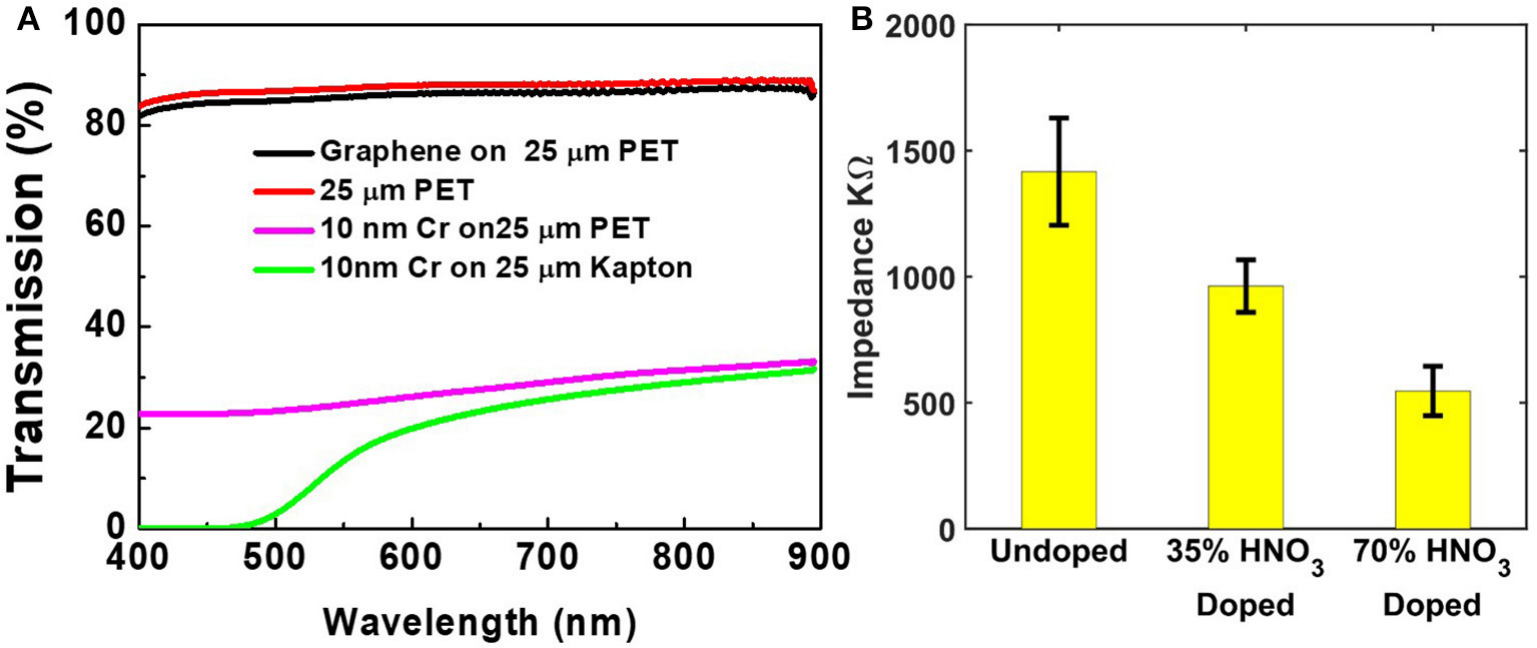
**(A)** The light transmission rate of different materials on PET and Kapton film for various wavelength. **(B)** Average impedance of arrays doped with nitric acid at different concentration. The data are the mean values and standard deviations of 16, 16, and 9 channels for the three methods respectively.

Transparent graphene arrays were fabricated on clear polyethylene terephthalate (PET) films. Different from polyimide substrates previously used for fabrication of graphene electrodes (Kuzum et al., 2014) colorless PET substrate allows high transmission across a broad range of wavelength of interest for optogenetics and multiphoton imaging. The transmission spectrum of bare 25 μm PET substrate and graphene on PET (Figure 1A) does not show any absorption peaks between wavelengths of 400 and 900 nm, while polyimide exhibits strong absorption below 500 nm. Figure 1A also demonstrates that even a metal films as thin as 10 nm significantly blocks light transmission, indicating the importance of using a transparent conductor for optogenetics or multiphoton imaging applications.

Toward fabricating high-yield large-area μECoG arrays, we first optimized the graphene transfer process to minimize the risks for crack formation and contamination. To this end, we have adopted and refined the “bubbling” transfer method (Wang et al., 2011; Gao et al., 2012), with which monolayer graphene grown on 20 μm thick copper foil with chemical vapor deposition (CVD) was transferred on PET substrate. First, poly(methyl methacrylate) (PMMA, 495 PMMA A4, Sigma-Aldrich) was spin-coated on top of the graphene/copper foil as the scaffold to support the monolayer graphene mechanically at a spin speed of 1,000 rpm for 60 s, which resulted in a thickness of 300 nm. The PMMA/Graphene/copper foil was connected to the cathode of a direct voltage source at 20 V, whereas a pair of metal tweezers was connected to the anode and immersed into a 0.05 M sodium hydroxide (NaOH) solution. When dipping the PMMA/Graphene/copper foil gradually into the solution, hydrogen gas bubbles were formed in between the graphene and copper layer because of electrolysis. These bubbles exfoliate the PMMA/graphene bilayer from the copper foil. The PMMA/graphene bilayer was then thoroughly rinsed by floating on the surface of deionized water for three times before placed onto the designed area. Bubbling-transfer method has a few advantages over conventionally used copper-etching method (Mattevi et al., 2011). First, bubbling-transfer does not require use of copper etchant, which contains FeCl_3_. FeCl_3_ attacks PMMA and makes PMMA removal much more difficult (Song et al., 2013). Second, since the copper foil is peeled off instead of being etched by part, there will be no copper particle residues left (Lin et al., 2012).

In addition to high yield, lowering the impedance of graphene electrodes is important to record neural activity with high signal-to-noise ratio. To this end, chemical doping with nitric acid has been proven to be an effective approach (Kasry et al., 2010; D'Arsié et al., 2016). Pristine graphene has a low concentration of charge carriers near Dirac point due to its unique band structure, whereas radicals in nitric acid, ${\text{NO}}_{2}^{0}$ and ${\text{NO}}_{3}^{0}$, decrease the Fermi energy level and hence increase hole concentration. Figure 1B shows a clear trend that the average impedance of graphene electrodes decreases if doped with higher concentration nitric acid for the same time (30 s). However, 70% nitric acid attacks PET substrate and significantly shortens the life span of the array, even though the initial result has the lowest average impedance. Therefore, 35% nitric acid doping was adopted in this work.

**Figure 2:**
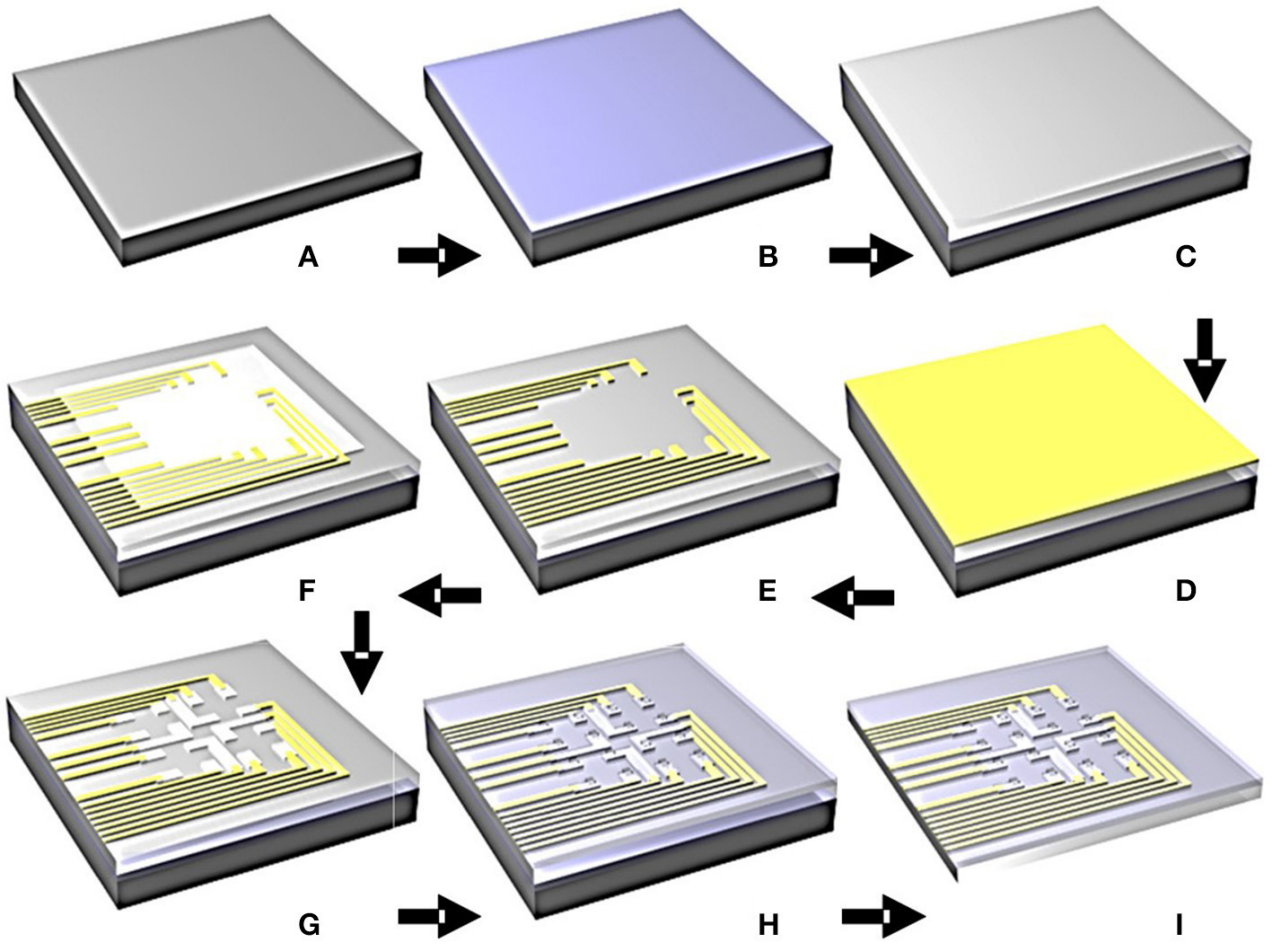
Fabrication processes of the flexible transparent graphene microelectrode array. **(A)** Cleansed silicon wafer; **(B)** PDMS adhesive layer; **(C)** PET film applied on PDMS layer; **(D)** Cr/Au sputtering; **(E)** metal wires patterned with UV-lithography and wet-etching; **(F)** graphene transferred by bubbling-method; **(G)** graphene contacts pattern with UV-lithography and oxygen plasma etching; **(H)** SU8 encapsulation; **(I)** array peeled off from the PDMS/silicon wafer.

**Figure 3:**
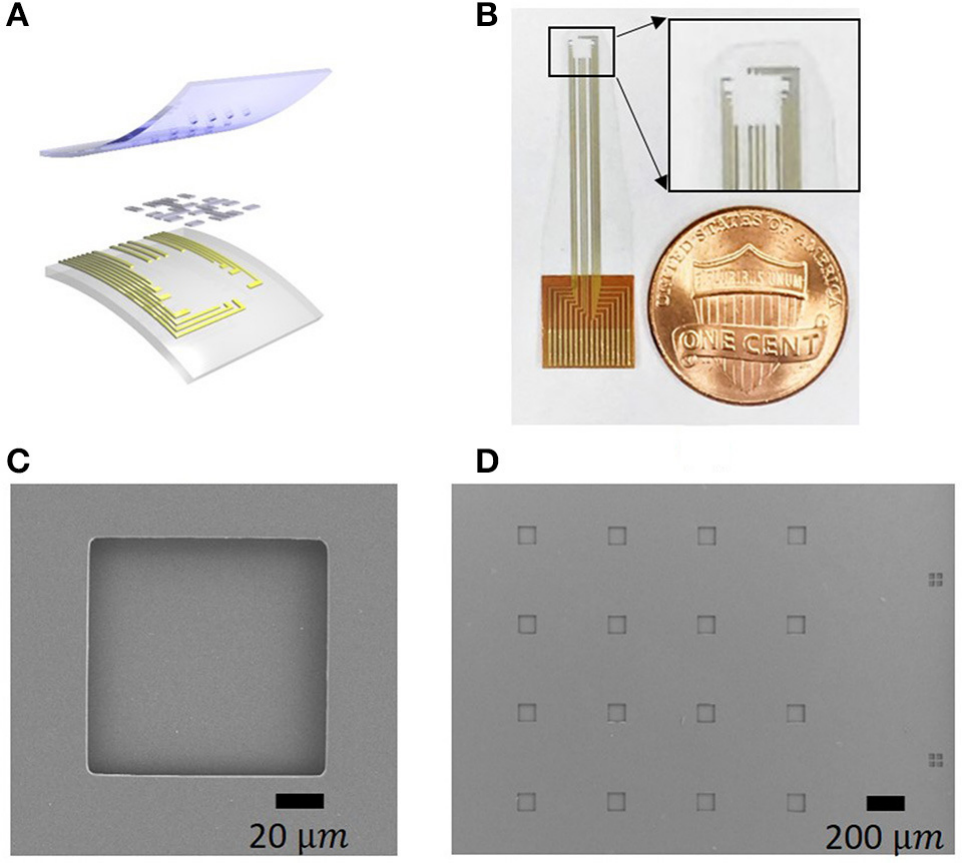
The structure and characterization of the transparent graphene electrode array. **(A)** The structure of the array consists of a PET film substrate, gold wires, graphene contacts, and SU8 encapsulation. **(B)** A photo of the flexible array. The inset shows the transparency of the array. A Scanning Electron Microscope (SEM) image of one channel **(C)** and the whole array **(D)**. Alignment marks are also included in **(D)** on the right side.

The graphene array fabrication started with coating a silicon wafer with poly (dimenthylsiloxane) (PDMS) as the adhesive layer to use as a rigid substrate carrier during the subsequent fabrication steps (Figures 2A,B). In this step, a 10:1 ratio of PDMS to curing agent was used. PET substrate was then placed on the PDMS layer (Figure 2C). Next, 10 nm chromium and 100 nm gold were sputtered on the PET substrate (Figure 2D). Metal wires were patterned with photolithography and wet-etching processes (Figure 2E). The etchants used in this experiment are Gold Etchant TFA and Chrome Etchant 1020 (Transene Company Inc.). Monolayer graphene was then transferred with the “bubbling” transfer method as described above (Figure 2F). Graphene electrodes were patterned with photolithography and oxygen plasma etching (Figure 2G). Finally, an 8 microns' thick SU8 layer was spin-coated and patterned with photolithography as the encapsulation layer. As shown in Figure 2H, only graphene electrodes were exposed, whereas everything else on the array was encapsulated. Each opening is a square with a side length of 100 microns. PET substrate was peeled off from the silicon wafer (Figure 2I). The final structure is shown in Figures 3A,B. The structural integrity of the arrays was examined by scanning electron microscope (SEM), as shown in Figures 3C,D for an individual channel and the whole array respectively. SEM images shows that the encapsulation and openings are patterned in the way we designed, where the openings are squares with 100 μm side length.

**Figure 4:**
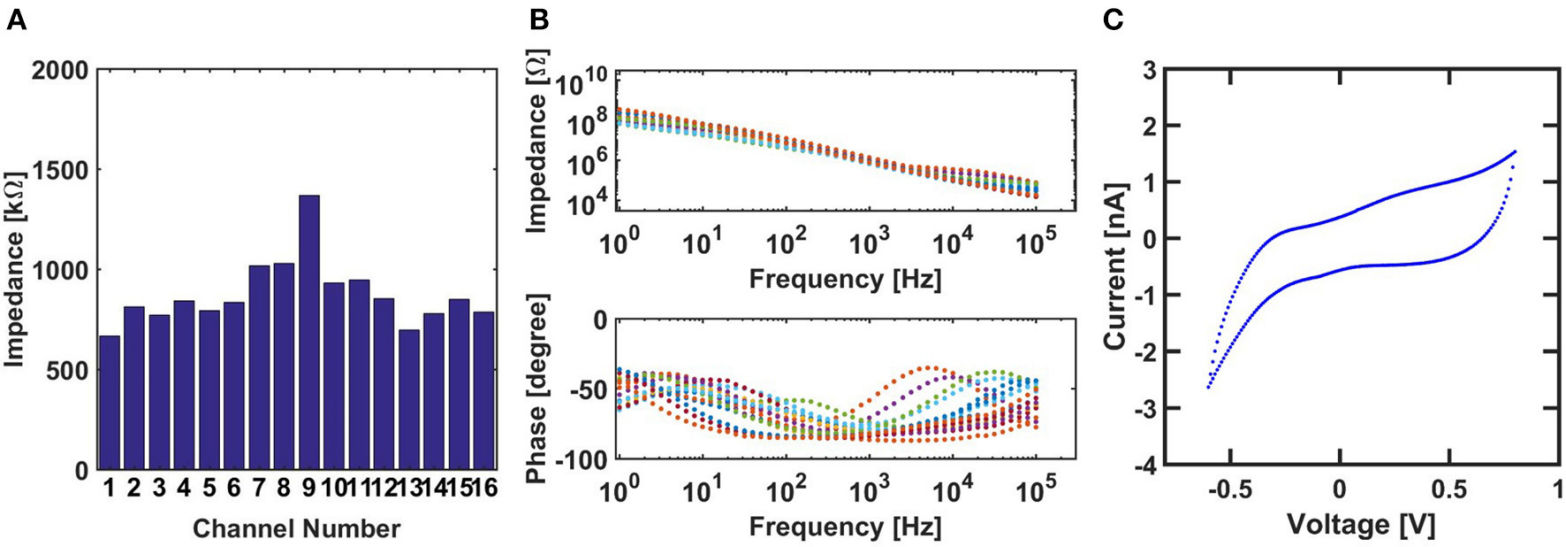
**(A)** Impedance distribution of all 16 electrodes of the array at 1 KHz. **(B)** Electrochemical impedance spectroscopy (EIS) of the 16 electrodes. **(C)** Cyclic voltammetry (CV) of a typical electrode of the array.

We characterized the arrays with Gamry Reference 600 plus using three-electrode configuration in 0.01M phosphate buffered saline (PBS). Platinum was used as the counter electrode, and Ag/AgCl as the reference. Electrochemical impedance spectroscopy (EIS) from a representative array is shown in Figure 4B. The upper and lower panel respectively, shows the modulus and phase of the impedance over the range from 1 Hz to 100 kHz. The 16 channels on this array have an average impedance of 872 KΩ at 1 kHz as shown in Figure 4A. Impedances higher than 3 MΩ is not desirable for electrophysiological recordings since they cause high noise and low signal-to-noise ratio. Cyclic voltammetry (CV) shown in Figure 4C is one the typical working channel, the shape of the curve indicates that the electrode is primarily capacitive.

## Results

### Artifact-free combination of optogenetics and electrophysiology

**Figure 5:**
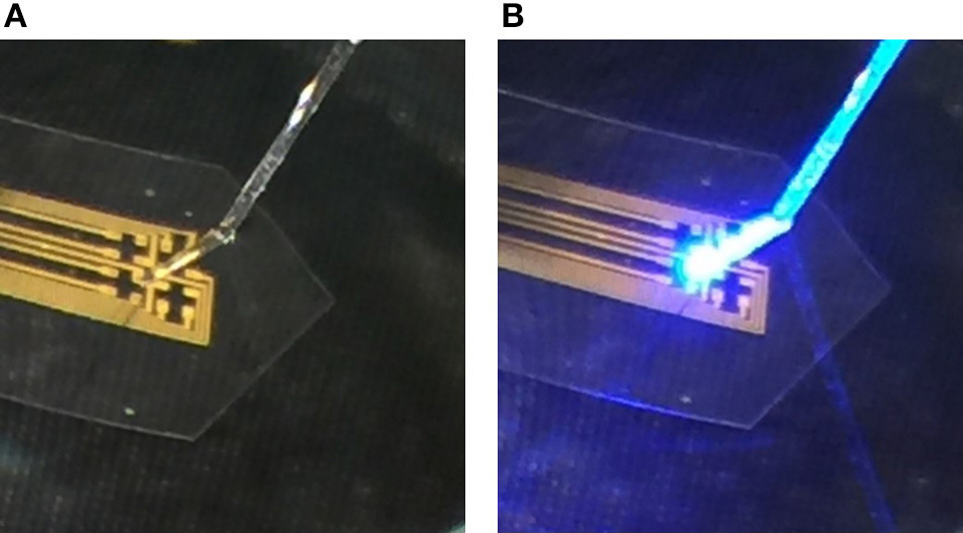
The pictures of artifact test for Au electrode with **(A)** LED stimulation off and **(B)** LED stimulation on.

One major problem for applications using simultaneous optical stimulation and electrical recording is the light-induced artifacts due to the photovoltaic (Becquerel) effect and the photothermal effects generated at the electrode-tissue interfaces (Cardin et al., 2010; Wu et al., 2013; Laxpati et al., 2014). For spike recordings, these light induced artifacts may be removed by filtering since spikes occur at a much higher frequency band (1–10 kHz) than the artifacts (1–100 Hz) (Han et al., 2011). However, in the case of LFP recordings, these artifacts can distort the recorded signal in both the time domain and the frequency domain (Han et al., 2009; Ozden et al., 2013). Thus, eliminating these light-induced artifacts is critical for neural applications that involve optogenetics and electrophysiology. In order to better understand the potential effect of light-induced artifacts in neural recordings, we first investigated them in simultaneous electrical recording and optogenetics experiments for conventional metal microelectrodes and then compared with graphene electrodes. For optogenetics, one of the commonly opsins is channelrhodopsin-2 (ChR2) which has a peak activation wavelength of 470 nm (Yizhar et al., 2011). The power intensity of the optogenetic stimulation for *in vivo* experiments are usually limited below 75 mW/mm^2^ with the pulse duration between 0.5 and 50 ms to prevent tissue heating and damage due to light absorption (Cardin et al., 2010). Therefore, 470 nm blue light with power intensity below 75 mW/mm^2^ and pulse duration between 10 and 100 ms were used to investigate the light induced artifacts for Au and graphene microelectrodes. The light stimulation was generated from a LED Driver (Thorlabs M470F1) and delivered to the electrode site through a 200 μm fiber. A standard optical power meter was used to measure the power intensity at the fiber tip over the electrode spot. Simultaneous electrical recordings were performed using Intan RHD2000 Evaluation System. The picture of the fiber and electrode with LED on and off is shown in Figure 5. The impedance of the Au electrode measured by Intan Evaluation System at 1 kHz frequency was around 300 kΩ. The impedance for the graphene electrode measured under the same condition was approximately 1 MΩ. During the experiment, the electrode was immersed in 0.01 M phosphate-buffered saline (PBS) solution.

**Figure 6:**
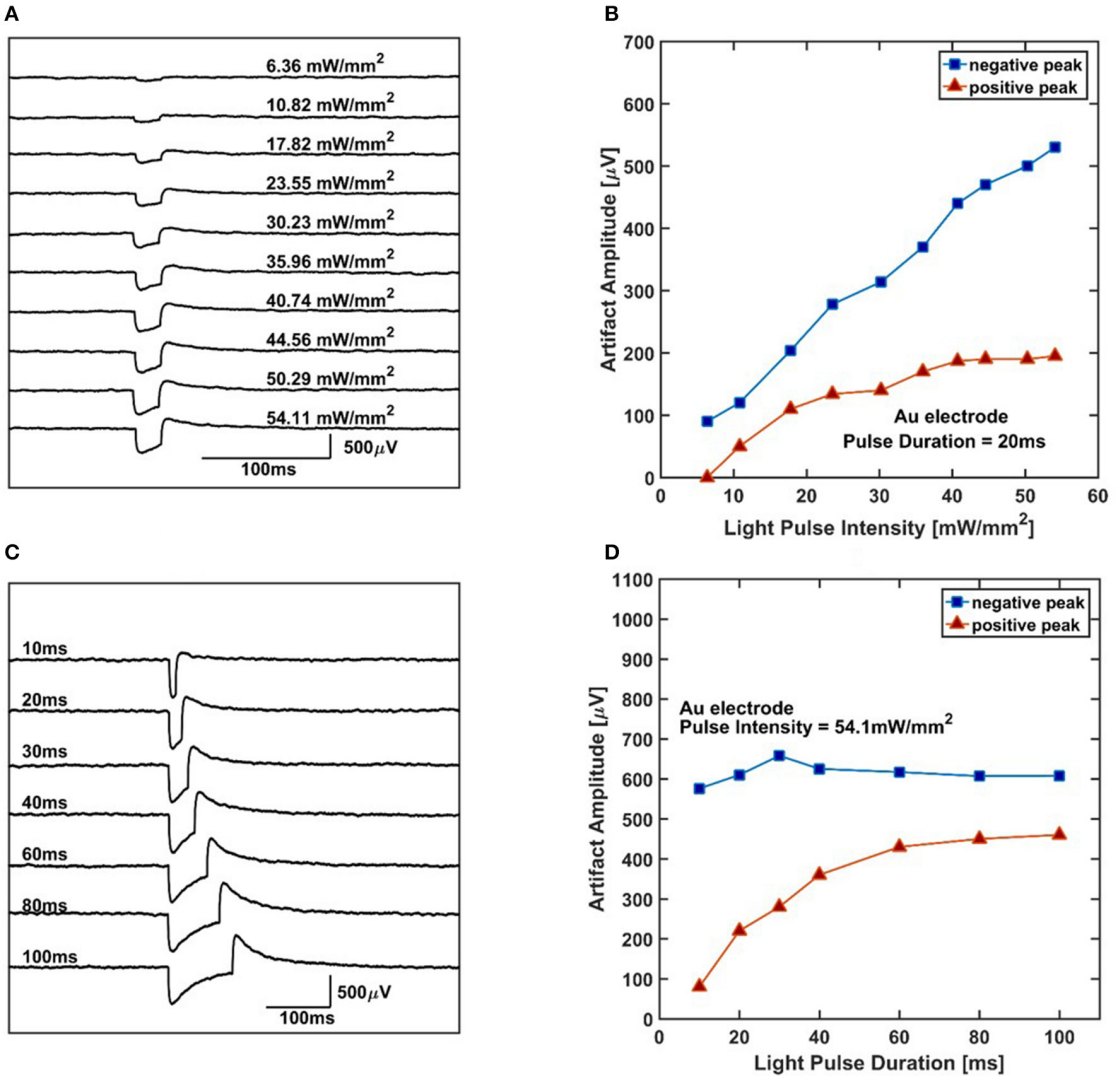
Artifact amplitude of Au electrode. **(A)** The typical recording artifact of one Au electrode for different light power intensities under 20 ms pulse duration. **(B)** The amplitude of negative and positive peaks of the artifact measured for different light power intensities. The result shows the negative peak amplitude is increasing linearly with respect to the light pulse intensity. The positive peak amplitude increases at first and then goes to saturation with enhancing light intensity. **(C)** The typical recording artifact of one Au electrode for different duration under 54.1 mW/mm^2^. **(D)** The amplitude of negative and positive peaks of the artifact measured for different durations. The result shows the negative peak amplitude almost remains constant with respect to the light duration. However, the positive peak artifacts increase as duration gets longer and saturate for large light durations.

Au electrodes exhibit prominent light-induced artifacts during optogenetic stimulation. Figures 6A,B shows the typical signals recorded by Au electrodes for various light intensities with a fixed light duration of 20 ms. It can be seen that the direction of the artifact when the LED turns on is negative and the amplitude grows almost linearly with the pulse intensity ranging from 5 to 54.5 mW/mm^2^. When the LED turns off, a positive peak shows up and decays exponentially. The amplitude of the positive peaks also gets larger as the light intensity increases. Figures 6C,D shows the typical signals recorded by Au electrodes for different light durations with a fixed light power intensity of 54.1 mW/mm^2^. When LED turns on, a negative peak shows up and its amplitude remains almost constant for different light durations. When LED turns off, a positive peak appears and its amplitude grows linearly in the beginning and saturates when the light duration approaches 100 ms.

**Figure 7:**
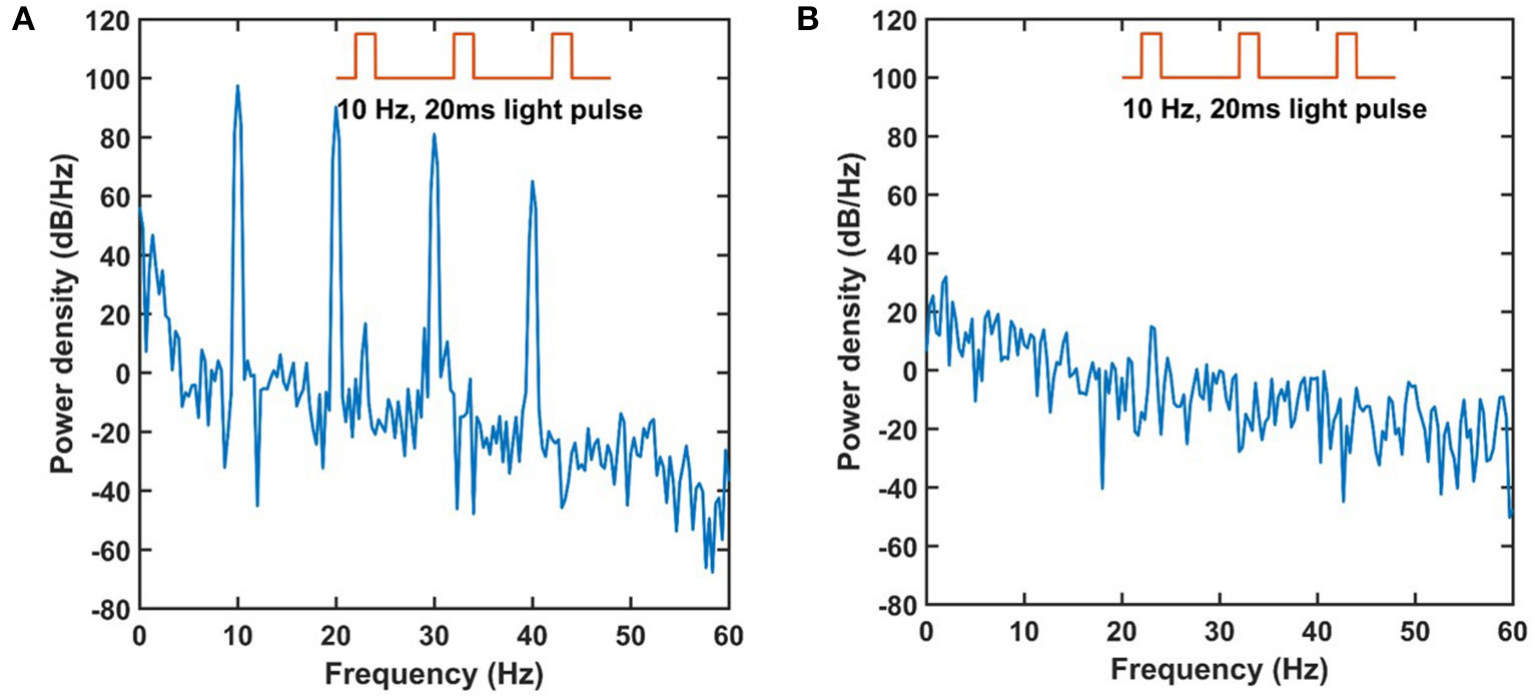
Power spectrum of artifacts recorded by the graphene and Au electrode. **(A)** The Au electrode records artifacts of 10 Hz and higher harmonic waves. **(B)** The graphene electrode shows no obvious artifacts corresponds to the light stimulation.

When the same experiments were repeated with graphene electrodes, no measureable artifacts were detected. To further inspect the artifacts of Au and graphene electrodes in the frequency domain, 10 Hz 20 ms duration light pulses were applied to the electrode site and the power spectrum was plotted for both recordings shown in Figure 7. For the Au electrode, there was an obvious 10 Hz peak corresponding to the artifact signals induced by the light stimulation of the same frequency. Besides the 10 Hz artifacts, some higher order harmonic signals, namely 20, 30, and 40 Hz, also existed in the recordings. For the graphene electrode, there were no detectable artifact components within the 0–60 Hz range. These experiments suggest that transparent graphene electrodes can be safely used in optogenetic stimulation and electrical recording experiments, guaranteeing crosstalk-free operation.

### Modeling light-induced artifacts in au electrodes

To explain the shape and behavior of the light-induced artifacts recorded by Au electrodes, we propose an equivalent circuit model (Supplementary Figure 1). In this equivalent circuit model, the controlled current source stands for the light-induced current generated at the Au electrode surface. The electrode-electrolyte interface can be modeled by the randles circuit which corresponds to the C1, R1, and Rs in the circuit model. Two input capacitors C2 and C3 are the built-in elements in the Intan electrophysiology chip, with the aim of removing the DC voltage signals generated at the electrode-electrolyte interface. The ADC samples the voltage signal on the resistor R2 which is filtered by the two input capacitors. Qualitatively, when the LED turns on, the initial voltage on the input capacitors are zero and the ADC samples a fast increasing voltage that corresponds to the negative peak. Then the voltage on the two capacitors increases due to continuous charging. This in turn causes the voltage that ADC samples decays toward zero exponentially. When the LED turns off, since the voltage on the capacitors can't change immediately, the ADC samples a positive voltage. Then again, the input capacitors discharge which causes the decay of the sampled voltage.

**Figure 8:**
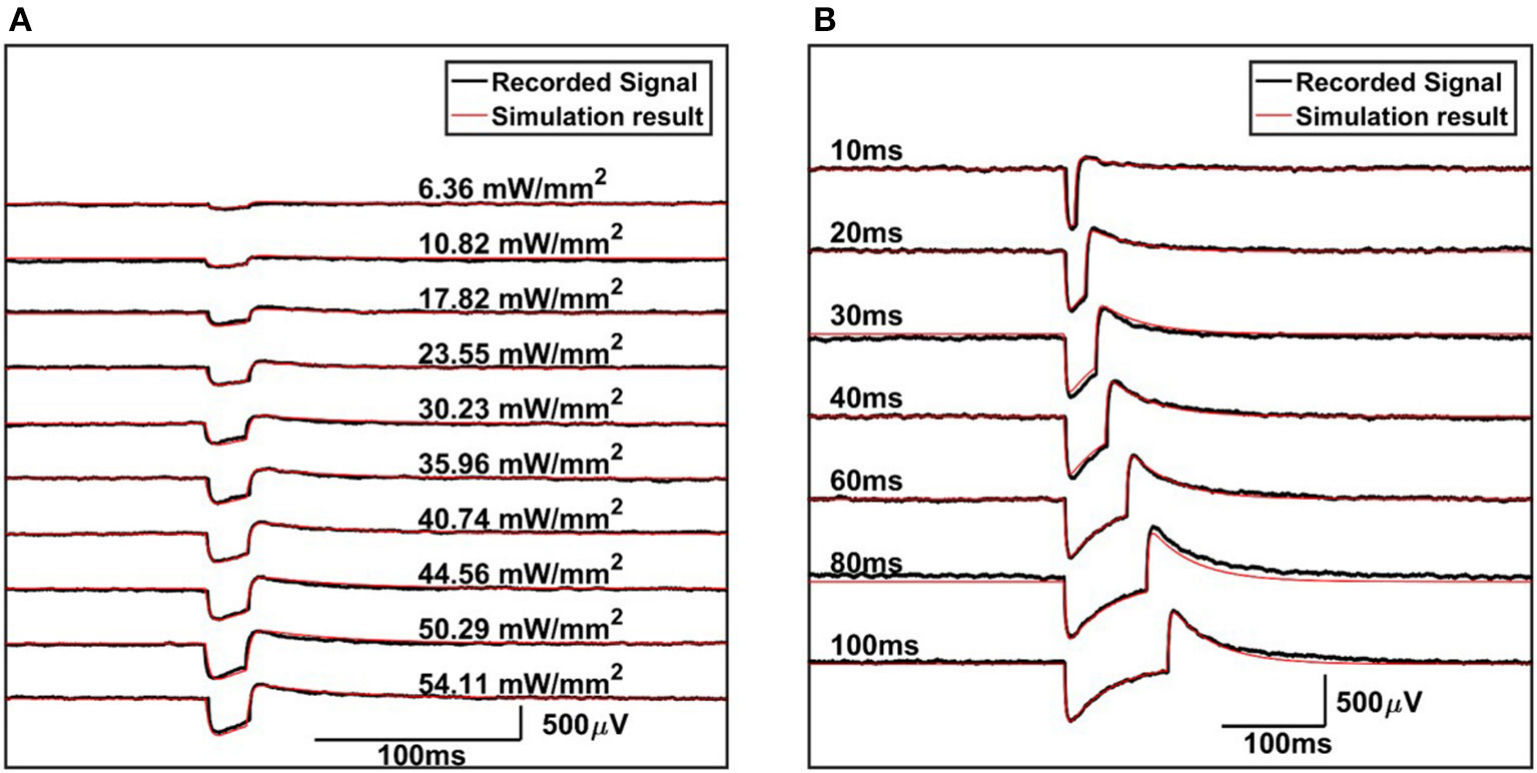
The comparison between the simulation results of the proposed circuit model and the experimental data for **(A)** fixed pulse width with increasing current source values and **(B)** fixed current source value with increasing pulse width.

To further validate our equivalent circuit model and quantitatively investigate the mechanism why the artifact is recorded, we formulated the problem as a parameter estimation task and tried to estimate the values of C1, R1, Rs and the current source from the experimental data. We chose to fit the voltage time series recorded under 54.11 mW/mm^2^ light power density with a light duration of 100 ms. First we performed Laplace transform to obtain an analytical expression of the voltage on R2 in the complex domain. Then we used inverse Laplace transform to get a function of the voltage on R2 in the time domain. At this point, the parameter estimation problem actually become a nonlinear least squares regression problem with the constraint of positive values for C1, R1, Rs and the current source. To solve for this regression problem, we added Lagrange multipliers to the least squares loss function and performed a gradient descent algorithm which run iteratively until convergence. With the estimated values plugged back into the circuit model, we run the simulations with different current source duration and amplitude. Here we assumed the light power density was proportional to the amplitude of the current source. The comparison between the simulation results and the experimental data is shown in Figure 8. It can be seen that the simulation result matches closely with the experimental data for all the different light durations and light power densities. These results confirm our proposed equivalent circuit model and explain why the light induced current grows linearly with the light power density.

### Optical design

In addition to the artifact-free operation enabled by graphene electrodes, optical design is also important for implementation of the closed-loop optogenetics. Insertion of the fiber tip into the cortical tissue has been used extensively since the first optogenetic experiments on animals (Gradinaru et al., 2007). LEDs coupled to waveguides have been demonstrated by a single group until now (Kwon et al., 2015) and was not a preferred method due to the incoherent nature of LED illumination pattern which makes it difficult to couple into waveguide structures. Also, the thermal effects associated with LED chips implanted on *in-vivo* probes have been a concern for many researchers (Kim et al., 2013; McAlinden et al., 2015; Wu et al., 2015; Kampasi et al., 2016). It was shown that the temperature rise could go up to 10°C (Kwon et al., 2015) and the laser diodes operated at a much higher power rating of 80 mW could only sustain it for a short amount of time (80 s) before the temperature threshold for safe limits (1°C above baseline tissue temperature) is exceeded on brain tissue (Kampasi et al., 2016). Thus, it is important to develop a system that keep the heat generating light source outside the body and guide the light toward the stimulation site via a waveguide structure.

Our optical design aims to deliver reliable optogenetic stimulation while achieving the following key conditions: external light source placement, minimizing the power consumption for the source and closed-loop operation that can respond to a given need of stimulation. For this purpose we used μLED chips that are small in size, sufficient in flux rating and require less energy compared to laser-based sources. Here we selected two different blue LEDs (460 nm) for our closed-loop optogenetics demonstration according to size and power considerations. First one is Cree DA2432 which operates at a high flux rating but has the drawback of greater lateral radiation profile. Second option, Cree TR2227 generates 33% less flux but has a well-defined Lambertian radiation pattern that promises a higher coupling efficiency. LED specifications can be found in Table 1.

**Table 1:** LED specifications.

**LED type**	**Dominant wavelength**	**Dimension**	**Flux rating**	**Max. forward voltage**	**DC forward current**
Cree TR2227	460 nm	220 × 270 μm^2^	21 mW	3.7 V	30 mA
Cree DA2432	470 nm	240 × 320 μm^2^	30 mW	3.4 V	100 mA

**Figure 9:**
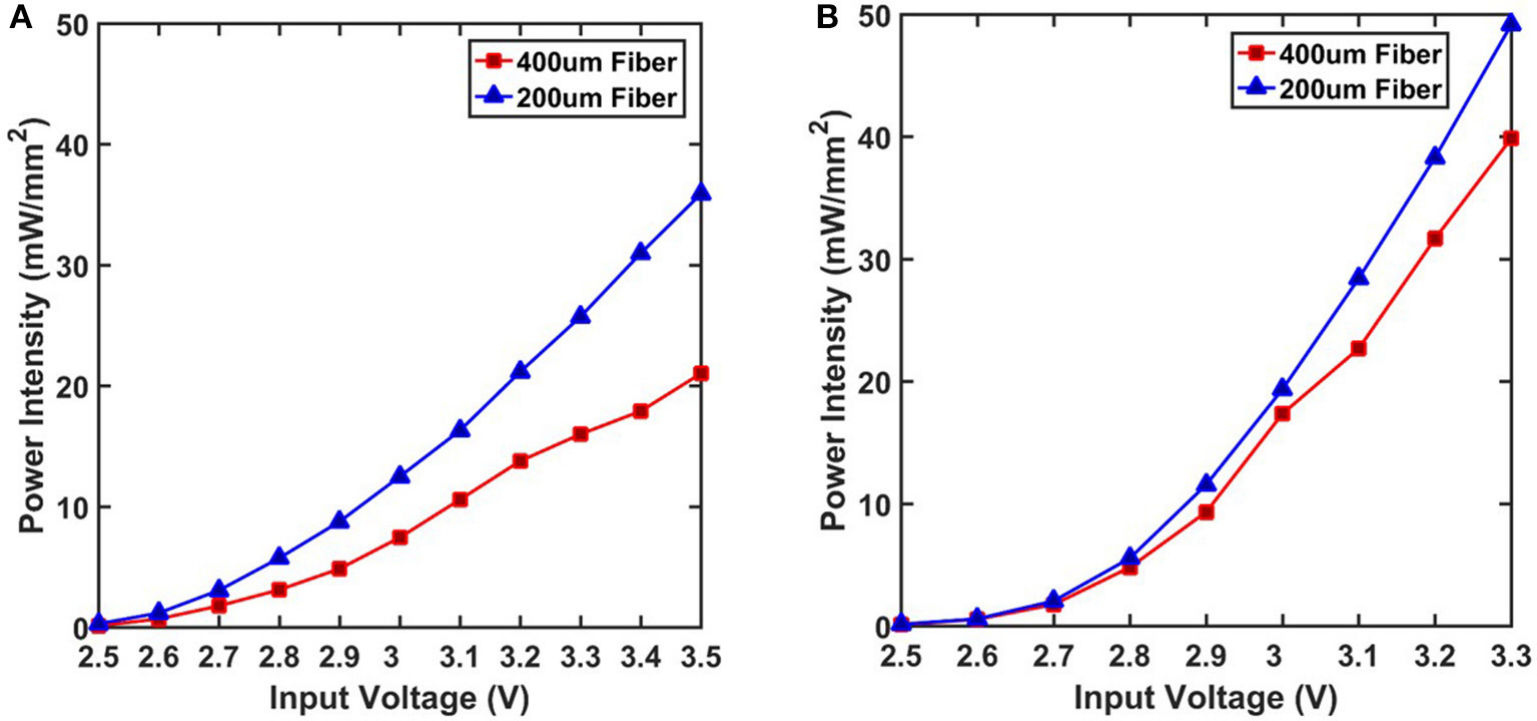
Total output power is measured at the tip of two fibers of different diameters after coupling. **(A)** Output from both fibers coupled with TR2227 LED chip. This chip has a greater maximum voltage rating and it was tested until 3.5 V. **(B)** Output from both fibers coupled with DA2432 LED chip. This chip reached the maximum allowed current at a lower voltage rating and was tested until 3.3 V.

The preparation of the optical setup consisted of soldering the LED chips and preparing bare fiber stubs to couple the light from LEDs into the target area. Cold solder was used to attach the LED chips on the Au contact pads on the flexible substrate connected to Au wires. The bare fibers were prepared by using a fiber stripping tool to remove the jacket as well as the thin cladding layer. This made it easier to cleave the fiber and achieve a flat surface for optimal coupling. The fiber stub was then positioned on top of the LED chip precisely using a micromanipulator tool. Emission from the LED is coupled into a bare fiber stub and the output power density from the fiber tip is measured by a photodetector (Figure 9). Ultimately, we achieved intensities of 40 and 50 *mW*/*mm*^2^ with the 400 and 200 μm fibers respectively, on the DA2432 chip. Likewise we observed 21 and 36 *mW*/*mm*^2^ with the 400 and 200 μm fibers respectively, on the TR2227 chip. These values are well above the required intensity of 1 *mW*/*mm*^2^ for optogenetic stimulation.

Besides the output power, the coupling efficiency between the fiber and the LEDs is also important for low energy consumption operation and effective stimulation. An important factor in achieving a strong coupling ratio between a source and a waveguide is how well the numerical apertures (NA) match. LEDs have incoherent radiation profiles that propagate in all directions and this makes it difficult to effectively couple their emission into waveguides. NA can be defined as the angle of emission/acceptance for a guided mode. As a source, LEDs effectively have high NA and a waveguide of comparable NA is required to maximize the coupling. The equation for angle of acceptance states that this is dependent on the refractive indices of the waveguide core, cladding and the surrounding medium.

$$NA=\;\sqrt{{n_{core}}^{2}-{n_{cladding}}^{2\;}}\;=n_{0}\sin\;\theta$$

**Figure 10:**
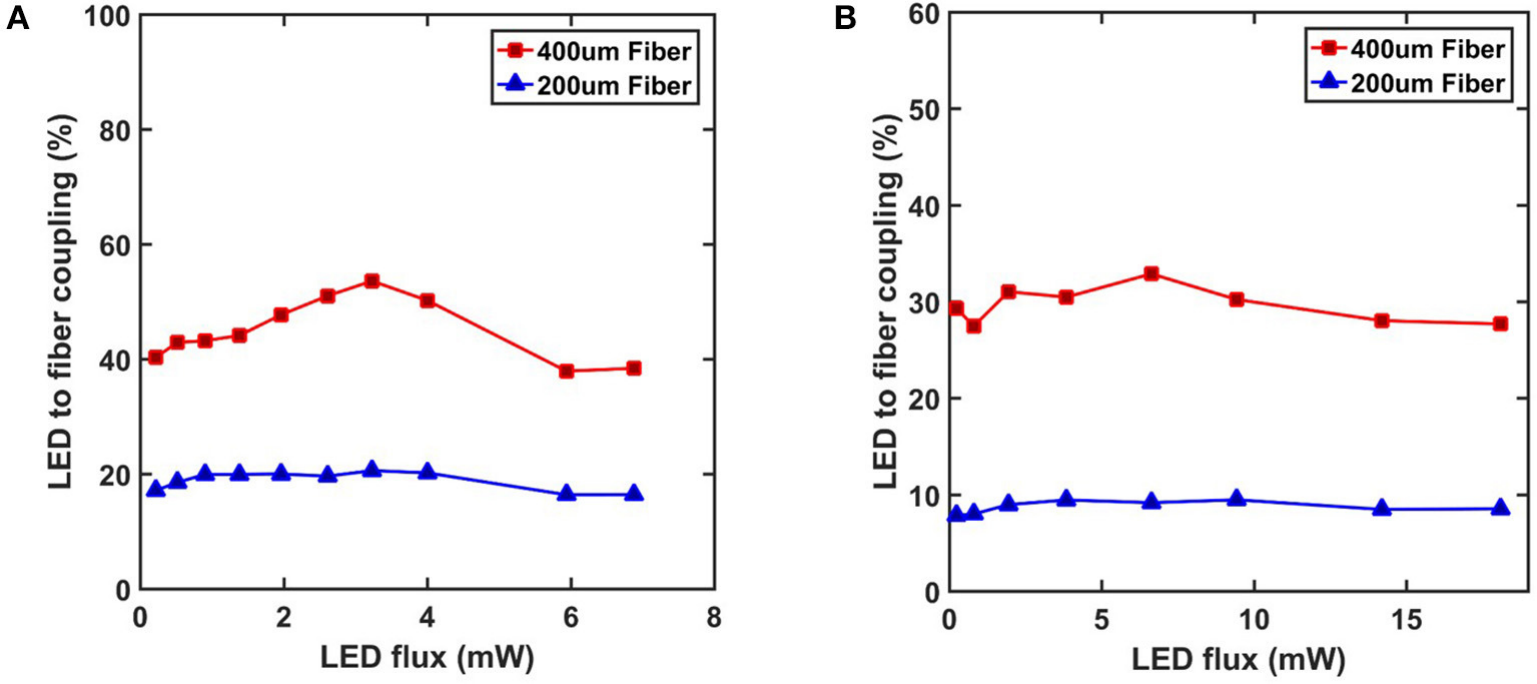
Coupling efficiency between fibers and **(A)** the TR2227 LED chip and **(B)** the DA2432 LED chip were calculated based on the data from Figure 9 and fiber dimensions. Higher efficiencies are observed at median flux ratings.

To explore the practical coupling between the LEDs and the fiber, two different fibers of 200/230 μm (core/cladding, Thorlabs FP200URT) and 400/425 μm (Thorlabs FP400URT) in diameters with a NA of 0.5 were tested. The comparison of coupling ratios is shown on Figure 10. The result shows that the radiation profile of the LED has a significant effect on the coupling. The maximum coupling efficiency for TR2227 was above 52% whereas the highest value attained by the DA2432 chip was only above 32% for the 400 μm fiber. A similar pattern was observed for the 200 μm fiber where the maximum efficiencies were 20 and 9% for the TR2227 and DA2432 chips respectively. The low coupling efficiency with the DA2432 chip is due to the side emission and the angle of acceptance of 30°, which could not cover the entire emission profile. The TR2227 chip did not suffer as much from this issue thanks to a radiation pattern that is almost a perfect Lambertian, maximizing the power coupled into the fiber's NA.

### Closed-loop system

The electrophysiology, such as EEG and ECoG, has fine temporal resolution to enable real-time monitoring of neural activities. The high specificity and millisecond scale temporal resolution of optogenetics offer fast control of targeted neurons (Deisseroth, 2011). These merits of the two technologies motivate the closed-loop modulations of specific neural populations, which facilitate the investigation of local neural circuit connections (Stark et al., 2014) or the interruption of seizures in freely moving animals (Krook-Magnuson et al., 2013; Pashaie et al., 2015). However, all closed-loop optogenetics systems demonstrated to-date use conventional metal-based electrodes which suffer from severe light artifacts that complicate the software signal processing algorithm, requiring a bulky system consisting of data acquisition instruments, computers, and light source controllers (Armstrong et al., 2013; Pashaie et al., 2015). For long-term studies in awake animals, a portable and small sized closed-loop optogenetics system is desirable to provide reliable recordings and localized optical stimulations.

**Figure 11:**
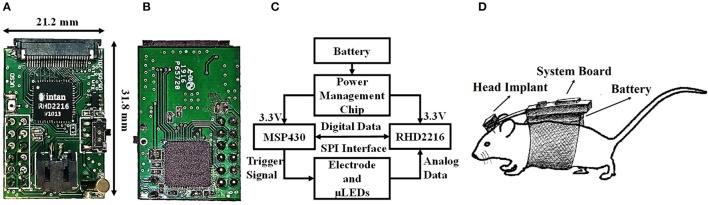
The closed-loop electrophysiology system. **(A)** The front side includes an Intan RHD2216 digital electrophysiology interface chip, power management chip, micro-switch, battery interface, and a 26-pin ZIF connector. **(B)** The back side locates the TI MSP430FR59891 microcontroller. **(C)** The diagram of the closed-loop system. **(D)** A schematic of the mice carrying the board.

Here, we present a portable low power consumption closed-loop optogenetics system to achieve real-time recording and light control of neural activities in behaving animals. The portable closed-loop system design is shown in Figures 11A,B. It consists of: (1) A MSP430 FR59891 microcontroller, (2) An Intan RHD2216 digital electrophysiology interface chip, (3) A 16 channel graphene micro-electrode and micro-LEDs, (4) A TPS781 (Texas Instruments) low-dropout (LDO) regulator, (5) A lithium ion polymer battery (Adafruit, 3.7 V, 150 mAh, 4.65 g, 19.75 × 26.02 × 3.8 mm). The board also has a backup serial port to facilitate data transmission to a computer for data visualization and storage. During the operation, as shown in Figure 11C, the microcontroller first configures the Intan chip through its built-in SPI communication interface. Then it drives the Intan RHD2216 chip to sample and convert the analog voltage detected on each channel of the micro-electrode under a certain sampling frequency (up to 30 kHz/channel). The digitized data are transmitted back to the microcontroller to implement closed-loop control algorithm. A simple LED driving circuit triggers μLEDs using pulse width modulation. Serial communication could also be used to transmit the recorded data back to the computer and facilitate visualization through the custom GUI software built using MATLAB (Supplementary Figure 2) if needed. However, closed-loop operation is tetherless and does not require the communication cable.

The electrodes are connected to the Intan chip directly through a ZIF connector. The μLED chip is soldered on a separate flexible flat cable and is driven by a simple transistor driver circuit, which is controlled by the microcontroller through pulse-width modulation. The brightness of the μLED is determined by the duty cycles of the pulses. The power management chip uses TPS781 series (Texas Instruments) as a steady power supply with ultra-low power consumption. It features two level of power supply, which can be used with the microcontroller to reduce the power consumption when necessary. The total weight of this closed-loop system is 11.3 g, which is acceptable for rodent experiments if a backpack configuration is used, as shown in Figure 11D. Further reduction of board weight and size could be achieved by employing smaller sized electronic components and higher energy density batteries.

The electrical and physical characterization of the closed-loop system is listed in Table 2. According to the MSP430 datasheet, the current consumption for the microcontroller is 100 μA/MHz for the active mode and 0.4 μA/MHz for the standby mode. Thus, for different applications, the power consumption of the microcontroller can range from 0.2 to 2 mW due to different system clock frequencies, the active/standby ratio and the operation status of the peripheral modules. From the datasheet of the Intan chip, the typical power consumption of RHD2216 under a sampling rate of 10 kHz/channel is ~5.5 mW. The current of the micro-LED used in the system is ~15 mA under 3.3 V input. If the average light-up duration of the LED is 20 ms per second, it will generate a power consumption of ~1 mW. Considering the efficiency of the power supply chip, the total power consumption is computed to be ~8 mW. Since the battery we use here is 600 mWh, the system is expected to work continuously for ~75 h under the conditions listed above. Depending on the specific RHD2216 sampling rate and the average LED light-up duration, the power consumption can vary significantly. For example, if the sampling rate drops to 1 kHz/channel and LED light-up duration 10 ms per second, then the battery can support up to 120 h.

**Table 2:** Electrical and physical characterization of the closed-loop system.

**Transparent graphene array**	**Board**	**Power supply**
16 channels	Sampling rate: 1–30 KHz	3.7 V Li Battery, ~75 h operation
Electrode dimension: 100 × 100 μm	Size: 21.2 × 31.8 × 1mm	Size: 19.75 × 26.02 × 3.8 mm
Total size: 4 × 4 mm	Weight: 6.65 g	Weight: 4.65 g

### Closed-loop operation using a threshold detection algorithm

**Figure 12:**
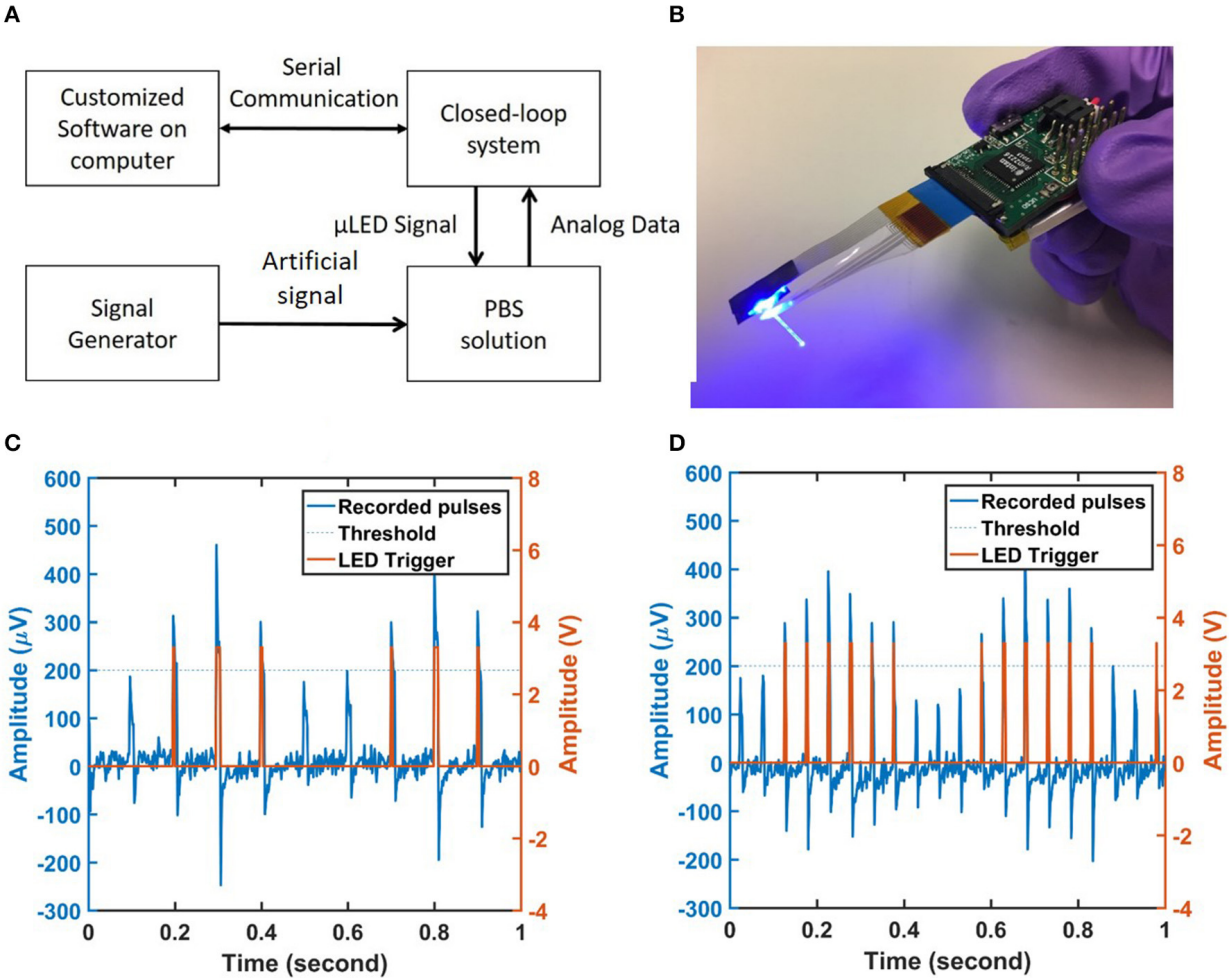
Testing setup of the closed-loop system. **(A)** The diagram of the testing setup. A signal generator was used to apply pulses with different amplitudes and duty cycles into the PBS solution. The analog data was sampled by the closed-loop electrophysiology system and transmitted to the computer for visualization using serial communication. All the recordings were performed in a properly grounded Faraday cage. **(B)** A picture of the working system. **(C)** Typical real-time recording data for one channel on the customized software built by MATLAB GUI App designer. A train of 10 Hz 10 ms duration pulses modulated by a 2 Hz sine wave was applied to the saline and the threshold was set to 200 μV. **(D)** The recorded signal on the software for a train of 20 Hz 5 ms duration pulses modulated by 2 Hz sine wave. The threshold remains the same.

The designed closed-loop system implemented a threshold detection algorithm and was tested under the framework shown in Figure 12A. If any of recorded channels of the graphene microelectrode array has an amplitude exceeding a certain threshold, then a LED trigger signal is sent out. Otherwise, the trigger signal was turned off. The picture of the closed-loop system with the graphene array and the fiber is shown in Figure 12B. We tested the closed loop operation using 10 Hz 10 ms width pulses and 20 Hz 5 ms width pulses. The amplitudes of both signals were modulated by a 2 Hz sine wave. The modulated signal was then applied to the 0.01 M PBS solution. The system sampled the signals of the electrodes and compared them with the predefined threshold stored in the microcontroller. To facilitate visualization, the recording data and the LED trigger signal were sent to a computer through the serial communication port and plotted in custom GUI software built using MATLAB GUI designer. The typical recording results are shown in Figures 12C,D. The threshold was set to be 200 μV. Modulated sine waves were also used to further test the performance of the system (Supplementary Figure 3). The peak-to-peak noise level of the recording system is shown in Supplementary Figure 4. The delay between the LED trigger signal and the effective recording signals can be in the scale of μs, depending on the sampling frequency. In this test setup, the sampling rate was chosen to be 1 kHz which indicated a maximum delay of ~1 ms. If the sampling rate was set to 30 kHz per channel which was the maximum allowed by the Intan RHD2216 chip, the total latency was measured as ~40 μs. These results show that the portable closed-loop system can be used for real-time control of various biological activities with minimum latency.

## Discussion

Neural circuits are complicated, nonlinear and nonstationary systems with dynamic changes in behavior on millisecond timescales. A robust and adaptive control method is required to achieve closed-loop optical control of such systems. Considering the safety and physiological constraints, the control system must be appropriate in most of the time-limited cases. Since closed-loop depends on real-time computation to keep up with rapid ongoing dynamics, there is always a computational budget that places limitations on the model complexity. Several different closed-loop control algorithms have been developed for optical modulation of the neural activity (Krook-Magnuson et al., 2013; Nguyen et al., 2014; Pashaie et al., 2015). Our proposed portable closed-loop system can support most of the feedback-control algorithms based on amplitude, frequency or power detection aiming different optogenetics applications.

One potential application of the closed-loop optogenetics is to bridge the horizontal disconnectivity caused by injuries in the cortical neural circuits. A closed-loop platform was proposed to force the level of neural activities in different recording sites to follow some predefined values (Pashaie et al., 2015). In the algorithm, the ECoG data was collected by the electrodes, preprocessed with a 10–150 Hz band-pass filter and a 60 Hz notch filter. Then the signal goes through a moving average filter which performs the integration to get the level of activity. This number is then compared with the predefined activity values to get an error signal. Then the error signal was translated into the pulse width of the optical stimulation to minimize the difference between the neural activity and the predefined set points. The translation is done via a proportional constant K_p_ which is learned by adjusting to different values through the calibration step.

Another possible application of the closed-loop optogenetics is to detect and control the neural disorders. A closed-loop seizure detection and control program was suggested to quickly identify and respond to the seizure onsets (Krook-Magnuson et al., 2013). The algorithm takes the real-time EEG recording data and extracts several features to help identify the seizures, such as signal power properties (amplitude correlation, fast/slow ratio, fast power drop), spike features (narrow spike, spike ratio), and the frequency properties (frequency band ratio). The amplitude correlation is calculated through a first order integrator. To get the fast/slow ratio, the DC component of the signal is removed and then two first order IIR filters are applied which act like a fast and a slow integrator respectively. Then the results of these two integrators are divided with each other to get the fast/slow ratio. The fast power drop is the output number of the fast integrator. The fast/slow ratio and the fast power drop are used to prevent a false trigger on a movement artifact instead of a seizure. The spike ratio is obtained by calculating an average distances between two consecutive spikes. This spike ratio and the narrow spike number are used to distinguish between the seizure activity and the regular repetitive small movements. To obtain the frequency band ratio, after removing the DC component of the signal, FFT transform is performed to the signal and the energy in two different frequency bands are calculated and then compared to respective thresholds. Finally, since the seizure features vary between individual animals, tuning operation is performed beforehand to achieve the thresholds for the above features. During the experiment, whenever the above criteria thresholds are crossed which means a seizure event was detected, the light stimulation is triggered randomly for 50% of the events.

The above algorithms can be implemented in the portable closed-loop system proposed here. The required low-pass, band-pass, and high-pass filtering can be implemented using the built-in analog and digital filters within the Intan RHD2216 electrophysiology chip. The integration, the rectification or the FFT transform can be implemented efficiently in the microcontroller through the Low Energy Accelerator (LEA) mode and the software DSP library. A flash memory chip may also be used to facilitate multi-channel high sampling rate signal processing tasks that are demanding for large storage resources.

In the future work, an ultra-low power MCU with larger RAM could be used to enhance the available computing resources for this closed-loop system. A digital signal processor (DSP) or FPGA could also be used as the control and signal processing unit to deal with intensive signal processing tasks, but this will definitely increase the power consumption. Wireless transmission can also be included in this closed-loop system to enable real-time visualization on a computer or other mobile devices, keeping the whole system tetherless in the meantime. Finally, specialized chip could be developed to integrate the electrophysiology sensing circuits and signal processing circuits so that the size and power consumption of the system could be further reduced. Note that there is always a power and computing trade-off, which should be considered for different applications and algorithms.

## Author contributions

XL designed the closed-loop hardware and software system. YL performed the fabrication and testing of the graphene electrodes. XL and YL investigated the light-induced artifacts for Au electrodes and the graphene electrodes. XL and YS established and validated the models for the light-induced artifacts in Au electrodes. EI designed the optical module of the closed-loop system. XL, YL, EI, and DK wrote the manuscript.

### Conflict of interest statement

The authors declare that the research was conducted in the absence of any commercial or financial relationships that could be construed as a potential conflict of interest. The reviewer CH and handling Editor declared their shared affiliation.
