# Supplementary material for: A Compact Closed-Loop Optogenetics System Based on Artifact-Free Transparent Graphene Electrodes
Source: Front Neurosci. 2018 Mar 6;12:132. doi: 10.3389/fnins.2018.00132 (PMC5845553; doi:10.3389/fnins.2018.00132)
Supplement: Supplementary file 1 [file DataSheet1.DOCX]

Supplementary Material

A Compact Closed-loop Optogenetics System based on Artifact-free Transparent Graphene Electrodes

Xin Liu^1^, Yichen Lu^1^, Ege Iseri^1^, Yuhan Shi^1^, Duygu Kuzum^1*^

*** Correspondence:** Corresponding Author: dkuzum@ucsd.edu

# Supplementary Figures
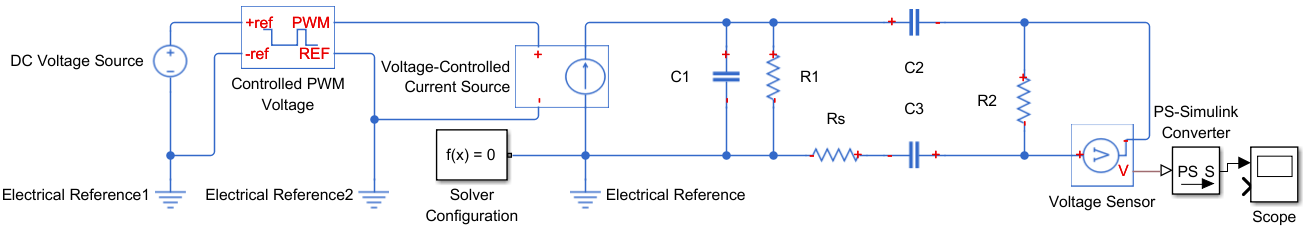


**Supplementary Figure 1.** MATLAB Simulink model to explain the artifact. The light pulses with different power intensity and durations are modeled as voltage controlled current sources. The electrode-electrolyte interface is modeled in the randles circuit (R1, C1, and Rs). The built-in capacitors of the ADC amplifier are modeled as C2 and C3. R3 stands for the input impedance of the amplifier. The recorded signal is just the voltage on R2.


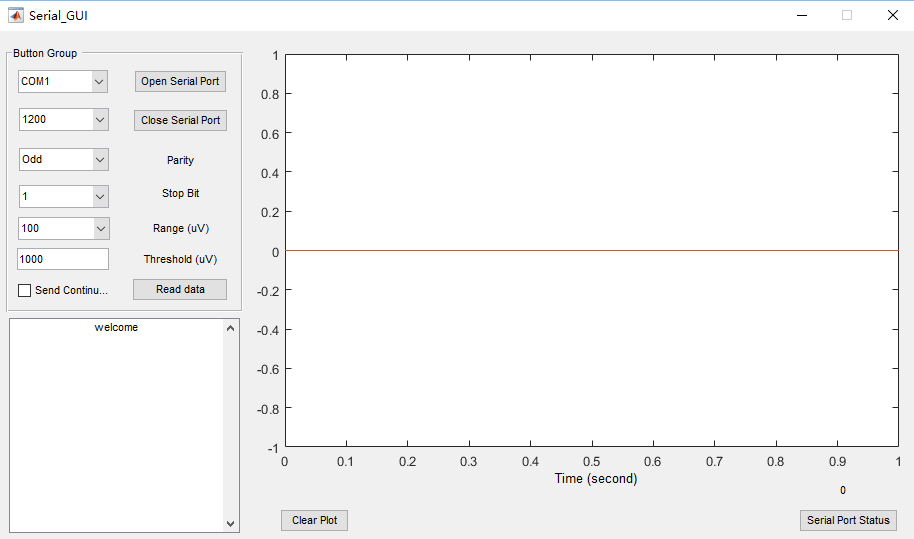


**Supplementary Figure 2.** Screenshot of the custom software built by MATLAB GUI designer. The software receives the recording data and the LED trigger status from the microcontroller through serial communication and then plot them in real time.


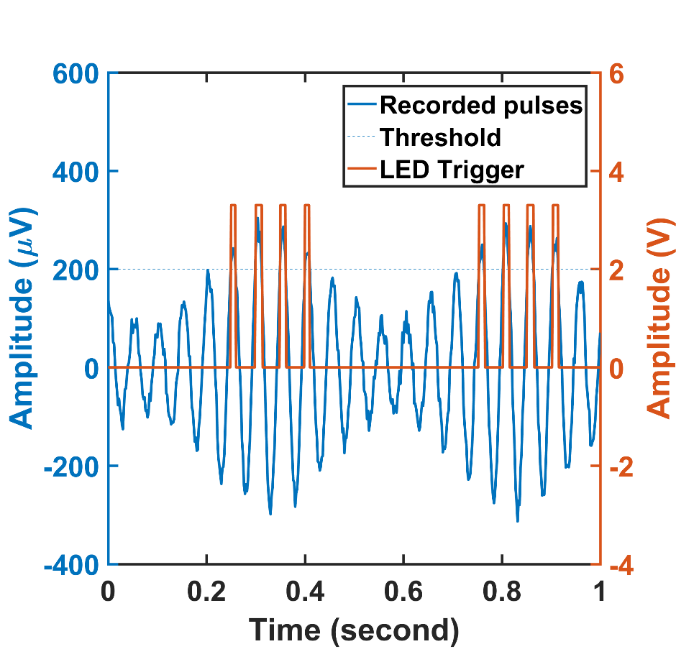

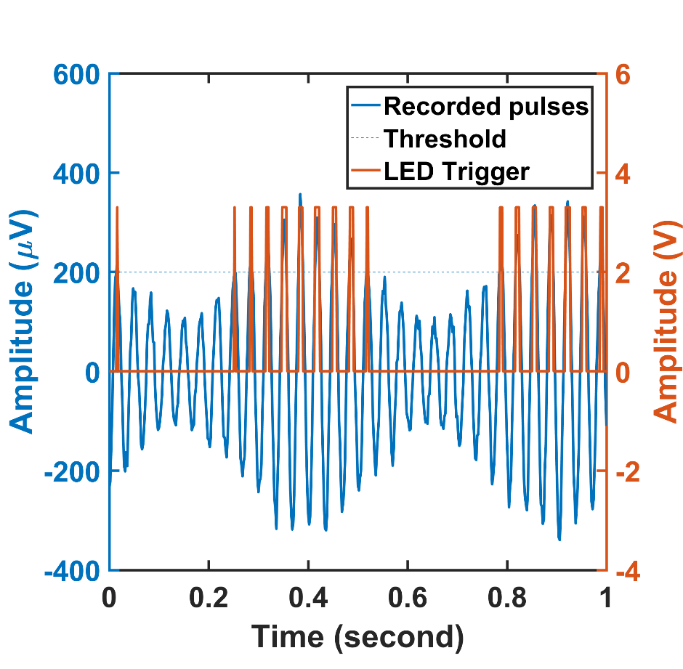


1. (b)

**Supplementary Figure 3.** The typical recording signals on the customized software for (a) 20 Hz sine waves modulated by a 2 Hz sine wave. The threshold is set to be 200 μV. (b) 30 Hz sine waves modulated by a 2 Hz sine wave. The threshold remains the same.


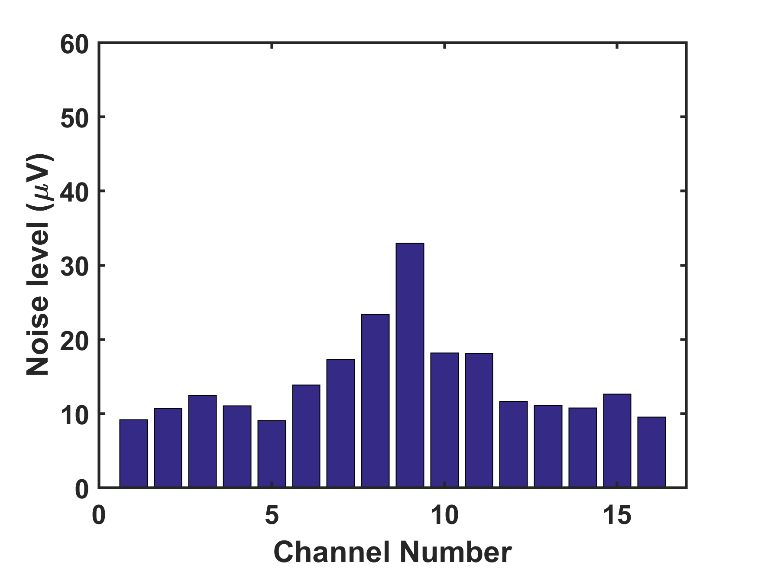


**Supplementary Figure 4.** The peak-to-peak noise levels of all the 16 channels during the bench test. The data are band-pass filtered between 10 Hz and 150 Hz.
